# Habitat Characteristics of Forest Fragments Determine Specialisation of Plant-Frugivore Networks in a Mosaic Forest Landscape

**DOI:** 10.1371/journal.pone.0054956

**Published:** 2013-01-24

**Authors:** Lackson Chama, Dana G. Berens, Colleen T. Downs, Nina Farwig

**Affiliations:** 1 Department of Ecology - Conservation Ecology, Faculty of Biology, Philipps-University of Marburg, Marburg, Germany; 2 School of Life Sciences, University of KwaZulu-Natal, Pietermaritzburg, South Africa; Institut Mediterrani d´Estudis Avançats (CSIC/UIB), Spain

## Abstract

Plant-frugivore networks play a key role in the regeneration of sub-tropical forest ecosystems. However, information about the impact of habitat characteristics on plant-frugivore networks in fragmented forests is scarce. We investigated the importance of fruit abundance, fruiting plant species richness and canopy cover within habitat fragments for the structure and robustness of plant-frugivore networks in a mosaic forest landscape of South Africa. In total, 53 avian species were involved in fruit removal of 31 fleshy-fruiting plant species. Species specialisation was always higher for plants than for frugivores. Both species and network-level specialisation increased with increasing fruit abundance and decreased with increasing fruiting plant species richness and canopy cover within fragments. Interaction diversity was unaffected by fruit abundance and canopy cover, but increased slightly with increasing fruiting plant species richness. These findings suggest that especially the availability of resources is an important determinant of the structure of plant-frugivore networks in a fragmented forest landscape.

## Introduction

Increasing impact of human land-use changes the structure and composition of ecosystems across the globe [1], [2]. Forests ecosystems are threatened worldwide with less than 50% still intact [3]. Large-scale destruction of natural forests leads to forest fragmentation. Small-scale changes within remaining forest fragments may alter the environmental conditions, e.g. the availability of fruit resources or vegetation structure [4], [5]. These changes may influence species diversity, and the overall functioning of forest ecosystems [1], [6].

The interaction among fruiting plants and frugivorous birds can be both antagonistic and mutualistic. The fruit handling behavior of the animals determines if these act as predators or as legitimate seed dispersers [7], [8]. Fruit or seed-eating animals profit from this interaction in the form of nutrition. Given that the seed passes handling by frugivores intact, plants profit from the interaction through dispersal of their seeds. Seed dispersal allows for an escape from the high density of competing siblings and from natural enemies in the vicinity of the parent trees [9]. Moreover, seed dispersal enables the colonization of vacant recruitment sites and directed dispersal to non-random habitats suitable for establishment [9]. Plant-frugivore interactions are particularly important in tropical and sub-tropical ecosystems as up to 90% of fleshy-fruiting plant species rely upon animal vectors to transport their propagules [9].

A number of studies have shown that forest fragmentation and degradation lead to changes in community composition of birds and in plant-frugivore interactions [10]–[13]. Thereby, the impact of fragmentation may depend on habitat characteristics of remaining forest fragments. Besides vegetation structure in general, a well-developed canopy stratum can be important for maintaining plant-frugivore interactions [14]. Further, the abundance and diversity of resources plays an essential role in sustaining bird communities within forest fragments. Both small- and large-bodied frugivores are attracted to forest fragments with high fruit abundance even across large distances [15]–[18]. Thus, a high availability of fruit resources and a well-developed canopy stratum may facilitate the persistence of diverse bird communities even within small forest fragments (e.g. [19], [20]).

Most of the studies investigating the impact of human activities on plant-frugivore relations studied interactions with single model plant species and yielded contrasting results [10], [11]. For instance, [10] showed reduced frugivore numbers and seed dispersal of a tropical tree as a consequence of anthropogenic fragmentation of a rainforest in Tanzania. In contrast, interactions of frugivores with the tree species *Prunus africana* were positively affected by human disturbance in a Kenyan forest [11]. The high variability in the results reveals the difficulty of using single species studies for predicting consequences of human impact on ecological processes. Thus, community-wide studies can contribute to a better understanding of plant-animal interactions in the face of land-use changes.

Recently, the analyses of complex interaction networks have gained in importance [21], [22]. These community-wide studies consider both occurrence and frequency of interactions between all species pairs within a community [2], [22]. The structure of interaction networks can be used to analyze the response of species relationships to land-use change. Low levels of specialisation of frugivores on plants, which can often be found in plant-frugivore interactions [23], may contribute to the persistence of networks, even if some interactions disappear [24], [25]. Moreover, a high diversity of interactions may lead to increase the robustness of the networks towards land-use changes. However, until now, the impact of land-use changes on entire plant-frugivore networks has rarely been studied (but see [14], [26]). Further, to our knowledge, no study has so far investigated the effects of habitat characteristics on the stability and robustness of plant-frugivore networks in a fragmented landscape.

Here, we present a study on the effects of fruit resource availability and canopy cover within forest fragments on plant-frugivore interaction networks in a fragmented sub-tropical forest landscape of South Africa. We assessed (1) overall levels of specialisation within networks in different forest fragments (2) the influence of resource availability, i.e. fruit availability and the diversity of fruiting tree species, as well as of canopy cover on the specialisation, interaction diversity and robustness of these networks. We expected to find (1) overall low levels of specialisation especially of frugivore species and (2) that high resource availability and a dense canopy cover of forest fragments lead to a higher interaction diversity and robustness of plant-frugivore networks.

## Materials and Methods

### Study Site and Design

We conducted this study over two successive years, 2009/10 and 2010/11 in a heterogeneous scarp forest landscape in and around Vernon Crookes Nature Reserve (VCNR) situated on the south coast of South Africa’s KwaZulu-Natal Province (150–610 m a.s.l., 30°16′ S, 30°35′ E). The necessary research permits for VCNR were obtained from Ezemvelo KZN Wildlife. All study sites outside VCNR were on private property owned by G. Archibald, who granted us access to his land.

Monthly rainfall in the area ranges from 1–148 mm and annual temperature from 6–31°C [27]. Covering an area of about 2,189 ha, VCNR is mainly a combination of hilly grasslands with wooded valleys. It is surrounded by a matrix of commercial sugarcane monocultures and timber plantations, within which a series of remnant natural scarp forest fragments exist. Fruiting by fleshy-fruiting plants occurs almost all year round, although the main fruiting season ranges from November to April, during the main rainfall season [28].

We worked in forest fragments that are representative for the area, i.e. large natural forest, forest fragments occurring naturally and being completely surrounded by natural grassland as well as remnant forest fragments embedded in a matrix of sugar cane fields at the border of VCNR. All forest fragments harbour similar communities of plants and birds and occur within the same natural geographic range of scarp forests [29], [30].

We established nine (200 m×200 m) study sites within forest fragments. The distance between study sites ranged from 0.53 to 1.06 km (mean = 0.80±0.3 km SD). Given the likelihood of strong edge effects in smaller forest fragments compared to the large fragments [31], we situated all sites near forest gaps or edges.

### Measuring Local Forest Fragment Characteristics

We measured habitat characteristics of local forest fragments by assessing, fruit abundance and fruiting plant species richness as well as canopy cover in each study site. We calculated fruit abundance (at the onset of fruit ripening) by estimating the number of fruits for each plant monitored and any other fleshy-fruiting plants within a radius of 50 m. We then calculated the mean fruit abundance for each study site, which ranged from 10,759 to 66,198 fruits (mean = 44,788±17,017 SD) over the two years. To determine fruiting plant species richness, we identified all fleshy fruiting woody plants in each site to species level. Fruiting plant species richness ranged from 9 to 15 (mean = 12±3 SD) across study sites. We visually estimated canopy cover standing in the center of four (50×50 m) quadrates in each site and calculated the mean per site. Canopy cover ranged from 64 to 92% (mean = 84±14% SD) across study sites.

### Assessment of Plant-frugivore Interactions

We observed all fleshy fruiting plant species in each study site to assess interactions with frugivorous birds. Observations were undertaken during the main fruiting seasons in 2009/10 and 2010/11. Within this timeframe, plant species were observed at their peak of fruit ripening. Due to the low abundance of plant species as well as the low abundance of fruiting individuals, only one individual of each plant species was monitored in each site. We observed each plant in each study site for a total of 18 h, ideally split into 9 h per year. In cases where we could not achieve 9 h in the first fruiting year, we increased the number of observation hours in the second fruiting year to attain the standard total of 18 h per plant. We split the observations into three monitoring sessions, namely early morning (06∶00 am –09∶00 am), mid-morning (09∶00 am to 12∶00 am) and afternoon (2∶00 pm –5∶00 pm), conducted at three different days during the main fruiting period of each plant species in each year. Observations of species were evenly spread across the three sessions. Using binoculars (Luger DA 10X42, Köln, Germany) observations were carried out from a camouflaged hide at ca. 20 m distance to the plant individual. All plant visiting birds and their fruit handling behaviour on the plant were recorded. If more than one feeding bird was present on the plant (<0.5% of observations), one randomly chosen individual was selected for which fruit consumption was observed.

### Network Analysis

We compiled interaction frequencies of each plant species (*p*) with each frugivore species (*f*) in a quantitative interaction matrix, whereby interaction frequency was defined as the number of fruit-eating visitors per plant species [32]. Thus, only species that fed on fruits (i.e., pecked on fruits or swallowed fruits, i.e. potential dispersers) were included in the analyses. Using the number of feeding visits to calculate the interaction frequencies between plants and frugivores allowed for comparison with other studies [14], [32]. We constructed interaction networks for each study site and calculated different network parameters at both the species and network level, which we used as response variables in our analyses. We quantified complementary specialisation in the sense of niche partitioning [33] as a measure of redundancy in our networks. At the species level, we calculated specialisation as the standardized Kullback-Leibler distance (*d'*) of each plant (*d'_p_*) and each frugivore (*d'_f_*) species as the deviation of the actual interaction frequencies from a null model assuming that all partners are used in proportion to their availability. We calculated a weighted mean of the index per site for both plants and frugivores [34]. In a similar way, we calculated *H_2_',* as the standardized two-dimensional Shannon entropy at the network level [34]. Both *d'* and *H_2_'* range between 0 and 1, for complete generalisation and complete specialisation, respectively [34]. We further calculated the diversity of all interactions in a network. Interaction diversity was computed with the help of the Shannon-diversity index. Finally, we calculated network robustness towards frugivore extinctions [35], [36]. In 1,000 iterations, frugivore species were randomly removed from each network and the number of non-dispersed plant species following this extinction was determined. The index of robustness then measures the area below this extinction curve and ranges from 0 to 1, with values close to 1 indicating high robustness of the network towards frugivore extinctions. The applied measure of network robustness does not take into account any changes in the realized niches of frugivores following the extinction of competitors. Thus, this index may underestimate robustness towards frugivore loss, as it represents a “worst-case”-scenario without plasticity of the remaining species. Further, different extinction scenarios, or different shapes of the extinction curve, are ignored by the robustness parameter used here. Yet, this simple measure can be seen as an approach to assess the impact of frugivore loss from the communities on plant-frugivore interactions. All network analyses were conducted with the bipartite package (version 1.13; [37]) in R (version 2.12.0; R Development Core Team). To test if annual variations affected the structure of our plant-frugivore interaction networks, we constructed separate interaction networks per study site for each of the two years. As observation hours were unevenly distributed between the two years on some plant species, we used a subset of species for which we had at least 6 h of observation per year. For species where more than 6 h of observations were available for each year, we equally sampled a 6 h-subset of the dataset from the three observation sessions. With the help of this sub-dataset, we analysed the differences in the above-mentioned network parameters between the two years. As year did not have an effect in any of the analyses (Table S1), we pooled the data set across the two years and used 18 h of frugivore observation for each plant species in each forest fragments. Henceforth, all analyses were based on this pooled data set.

### Statistical Analysis

All habitat characteristics, i.e. fruit abundance, fruiting plant species richness and canopy cover were uncorrelated (all p-values >0.423). We used a linear mixed effects model to test the effect of the above-mentioned characteristics as well as of trophic level (plants *vs.* frugivores) on species specialization (*d'*). Trophic level was nested within plot for this analysis. Moreover, we used fruit abundance, fruiting plant species richness and canopy cover as explanatory variables to examine their effect on complementary specialisation (*H_2_'*), interaction diversity and network robustness in separate linear models. All statistical analyses were performed in R using packages base and nlme. We further tested for a correlation of interaction diversity and network robustness.

## Results

Across the nine study sites, we monitored a total of 31 fruiting plant species (Table S2). During 1,854 observation hours we recorded a total of 53 frugivorous bird species and 8,284 frugivore visitors involved in fruit removal activities on the focal plants (Table S3). Plants that had most interactions with frugivores included the Common Wild Fig (*Ficus burkei*), Forest Knobwood (*Zanthoxylum davyi*) and Red-beech (*Protorhus longifolia*). Frugivores with most interactions on plants included the Dark-capped Bulbul (*Pycnonotus tricolor*), Cape White-eye (*Zosterops virens*) and Knysna Turaco (*Tauraco corythaix*) (Figure S1).

### Specialisation of Plants and Frugivores

**Figure 1:**
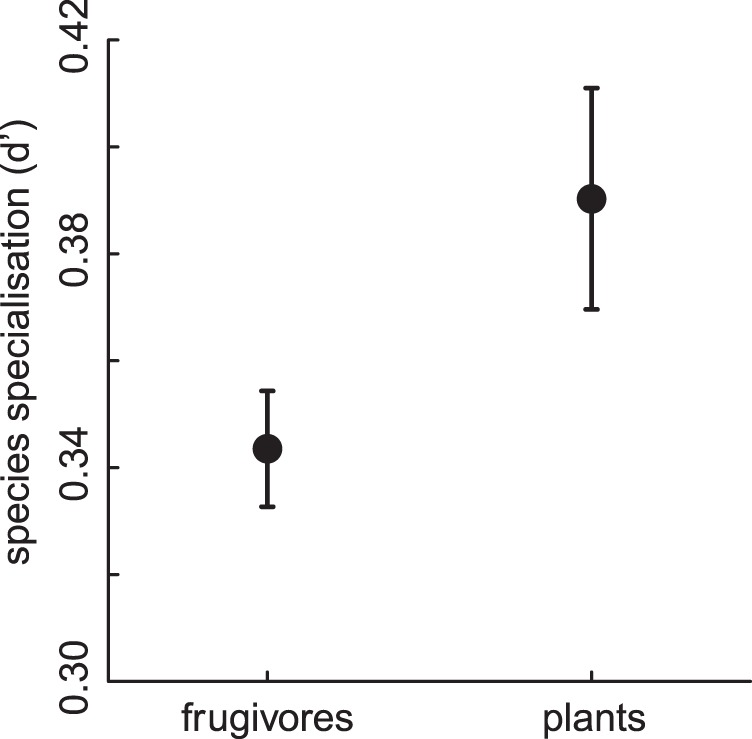
Mean ± SE specialisation of frugivores and plants within plant-fugivore networks. (n = 9).

The mean weighted species specialisation (*d'*) of the trophic levels within our networks was significantly higher for plants (0.39±0.02 SE) than for frugivores (0.34±0.01, Table 1; Fig. 1; Table S4). Species specialisation further increased with increasing fruit abundance, but decreased with increasing fruiting plant species richness and canopy cover (Table 1; Table S4).

**Table 1:** Effects of habitat characteristics and trophic level on the structure of plant-frugivore networks.

	species specialization (*d'*)		network specialization (*H_2_'*)		interaction diversity		network robustness	
	t	P	t	P	t	P	t	P
fruit abundance	3.26	**0.022**	4.20	**0.008**	1.08	0.329	−0.19	0.854
fruiting plant species richness	−3.13	**0.026**	−9.42	**<0.001**	2.21	**0.078**	1.88	0.119
canopy cover	−3.20	**0.024**	−3.72	**0.014**	1.34	0.238	−0.44	0.676
trophic level	3.74	**0.006**	–	–	–	–	–	–
R^2^	–		0.96		0.65		0.42	

Species specialisation (*d'*), network specialisation (*H_2_'*), interaction diversity and robustness of plant-frugivore networks (n = 9) in relation to fruit abundance, fruiting plant species richness, canopy cover (%) and trophic level (plant *vs.* frugivore). To investigate effects on species specialization, trophic level was nested within plot in a linear mixed effect model; effects on all other dependent variables were analysed using linear models. Given are *R^2^*, *t* and *P* values, if applicable. Note: all significant or marginally significant *P values* are highlighted in bold.

### Network Specialisation (H_2_'), Interaction Diversity and Robustness

**Figure 2:**
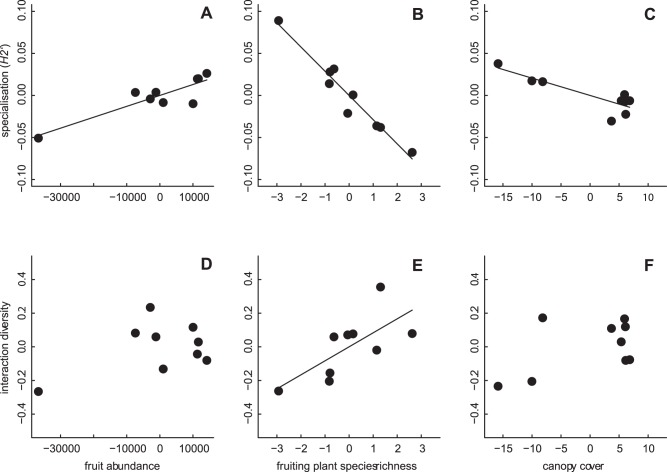
Effect of different habitat characteristics on network specialisation and interaction diversity. Network specialisation (*H_2_'*; A, B, C) and interaction diversity (D, E, F) in relation to fruit abundance, fruiting plant species richness and canopy cover (%), for plant-frugivore networks (n = 9; for calculation of network parameters, see methods). Shown are residuals of both x and y variables controlling for all other variables in the model (see Table 1).

Overall, the mean network complementary specialisation (*H_2_'*) was 0.44±0.02 (Table S4). Network specialisation increased with increasing fruit abundance (Table 1; Fig. 2a), and decreased with increasing fruiting plant species richness (Table 1; Fig. 2b) and canopy cover (Table 1; Fig. 2c; Table S4). The mean diversity of interactions in the networks was 3.54±0.07 and was unaffected by fruit abundance and canopy cover (Table 1, Fig. 2d, f; Table S4). However, interaction diversity tended to increase with increasing fruiting plant species richness (Table 1; Fig. 2e; Figure S1). The mean robustness of networks towards frugivore extinction was 0.48±0.01. Network robustness was unaffected by any of the habitat characteristics (Table 1; Table S4). Interaction diversity and network robustness were positively correlated (Pearson correlation, r = 0.78, p = 0.014).

## Discussion

Our study shows that specialisation of plants was higher than that of frugivores. Generally, the different habitat characteristics affected network structure but not its robustness to species extinctions. High fruit abundance led to higher specialisation, while increasing fruiting plant species richness and canopy cover reduced specialisation both at the species and network level. Interaction diversity slightly increased with fruiting plant species richness and was unaffected by fruit abundance and canopy cover.

### Network and Species Specialisation

Overall, network specialisation was comparable to other plant-frugivore networks (*H_2_'* = 0.18–0.51 [38]; *H_2_'* = 0.30 [14]). In comparison to e.g. pollination networks (median specialisation of 0.55 across 21 networks, [23]), rather low specialisation in plant-frugivore networks is generally expected in subtropical and tropical ecosystems [14], [39]. Especially in the tropics, year-round fruit availability and diversity and dependence of frugivores on these fruits are both high [40]. Thus frugivores forage in a generalist way, leading to low network specialisation in tropical ecosystems. This implies redundancy among the interactions within the networks, and therefore potentially a stability towards disturbances [41], [42].

Within our networks, plant species were on average more specialised than frugivores, and thus have a higher dependence on frugivores than vice versa. This asymmetry in mutual dependence is a common pattern in plant-frugivore networks [25], [43]. It may be caused by the fact that frugivores are the only mobile nodes within the networks, and are able to track and explore plant resources [15], [16]. Plants, in contrast, are sessile and depend on the frugivores’ choice and visitation. From the plantś perspective, the high specialisation on frugivores, and thus on potential seed dispersers, might make them vulnerable to the loss of bird species from the community. This could ultimately challenge their regeneration potential. In contrast, the lower specialisation of birds seems reasonable as the year-round dependence of tropical and subtropical frugivores on fruit resources leads to the consumption of a great variety of fruit species [40], [44], [45]. Further, several studies have shown that frugivore species are able to track new fruit resources across vast spatial and temporal scales [15], [16], [46]. Consequently, frugivores may be less prone to fluctuations in resource availability across the plant community.

### Fruit Abundance, Fruiting Plant Species Richness and Network Structure

An increase in fruit abundance resulted in an increase in specialisation at both network and species level. This implies that a higher availability of fruits within a forest fragment leads to higher specialisation of frugivores on plants and vice versa. The increase in specialisation has contrasting effects on the two trophic levels within the network. From the plants’ perspective, high specialisation may limit interactions with a broad spectrum of frugivores, thereby increasing their dependency on single or few frugivores that may either be effective or non-effective, e.g. based on their foraging capacity, efficiency (e.g. fruit handling behavior) and dispersal distances [47]. From the frugivore perspective, high resource availability reduces both the average foraging time as well as the distance that they have to cover between fruiting plants [48]. Moreover, this may also reduce competition for resources [23], allowing frugivores to access specific fruit types with less handling and search effort or energy loss [23], thereby increasing specialisation. In contrast, lower availability of specific resources increases generalisation due to high search effort, as confirmed by the negative effect of fruiting plant species richness on specialisation (see also [49]). Further, the increasing variety of available fruit resources allows for efficient balancing of nutrient diet, which again increases frugivore generalisation [40].

The increase in interaction diversity particularly with increasing fruiting plant species richness denotes that high diversity of food resources may promote network stability. In fact, we found a positive relationship between interaction diversity and robustness of our networks. Under such conditions, plants would profit from a broad spectrum of seed dispersing frugivores as this may reduce the clumping of seeds in the landscape. This may potentially aid the recruitment of their offspring in diverse communities. Nonetheless, neither fruit abundance nor fruiting plant species richness affected robustness at the network level. Changes of specialisation and interaction diversity along gradients of habitat characteristics are not reflected in varying robustness of the networks. This could be caused by the comparably high overall generalisation and redundancy within our networks, which makes the networks insensitive towards the loss of single frugivore species.

### Canopy Cover and Network Structure

Specialisation at both species and network level decreased slightly with increasing canopy cover, while interaction diversity and network robustness where unaffected. Decreasing specialisation with increasing canopy cover is congruent with results from a study in a Kenyan rainforest [14], which found that obligate frugivores, the frugivore guild which is potentially least specialised, are foraging mostly in the canopy stratum. Further, fleshy-fruiting plants are often aggregated under the canopy of tall trees, potentially due to a higher seed rain under these trees [40]. Canopy cover might actually represent the long-term level of resource availability within a fragment, while fruit availability may only be a snapshot of the resources available in a limited time span.Thus, the direct negative effect of canopy cover on specialisation might be caused by an indirect effect of a higher fruit crop and a higher attractiveness to frugivores with increasing canopy cover. High resource availability will, in turn, maintain a broad spectrum of generalist frugivores within the community. Yet, despite its effects on network specialisation, canopy cover neither influenced interaction diversity nor the robustness of plant-frugivore networks. Other habitat characteristics, e.g. vegetation structure and complexity, may thus be more important in promoting the maintenance of complex frugivore communities in the long-term.

### Conclusions

Our study shows that networks across local forest fragments were all characterized by redundancy in the associations between plants and frugivores, suggesting a stable coexistence of species in the plant-frugivore communities. Thus, secondary extinctions of mutualists, due to resource losses are unlikely in these networks [50], [51], as species have numerous reassembly pathways [52]. Especially the abundance and diversity of fruit resources seem to be a key factor driving specialisation of plant-frugivore networks. However, besides the habitat characteristics measured here, i.e. resource availability and canopy cover, other factors might be more important in determining the ultimate robustness of these networks. We particularly encourage conservation efforts to promote management strategies that will maintain habitat quality in remaining forest fragments. Moreover, future studies should also endeavour to establish how such complex interactions between plants and frugivores translate into effective seed dispersal and forest regeneration.

## Supporting Information

Figure S1 **Plant-frugivore interaction networks in nine scarp forest fragments in Kwazulu-Natal, South Africa.** Grey shades represent the frequency of interactions among frugivores and plants within a network, with black representing most frequent interactions.
(EPS)

Table S1 **Effects of habitat characteristics and season (year 2009/10, year 2010/11) on the structure of plant-frugivore networks.** Species specialisation (*d'*), network specialisation (*H_2_'*), interaction diversity and robustness of plant-frugivore networks (n = 9) in relation to fruit abundance, fruiting plant species richness, canopy cover (%) and season (2009/10 and 2010/11). A subset of the complete data set was used for these analyses. For each species where more than 6 h of observation in each year were available, we equally sampled a 6 h-subset of the dataset from the three observation sessions (see main text for details). Effects on all dependent variables were analysed using linear mixed effect models, with year nested within plot. To investigate effects on species specialization, trophic level was nested within year and plot. Given are *t* and *P* values. Note: all significant or marginally significant *P* values are highlighted in bold.
(DOCX)

Table S2 **Common and scientific names of fleshy-fruiting plants in nine plant-frugivore networks.**
(DOCX)

Table S3 **Species codes, common and scientific names of frugivores (birds) in nine plant-frugivore networks.**
(DOCX)

Table S4 **Measures of plant-frugivore networks and habitat characteristics of nine scarp forest fragments in South Africa.**
(DOCX)
