# Supplementary material for: Habitat Characteristics of Forest Fragments Determine Specialisation of Plant-Frugivore Networks in a Mosaic Forest Landscape
Source: PLoS One. 2013 Jan 24;8(1):e54956. doi: 10.1371/journal.pone.0054956 (PMC3554686; doi:10.1371/journal.pone.0054956)
Supplement: Table S1 — Effects of habitat characteristics and season (year 2009/10, year 2010/11) on the structure of plant-frugivore networks. Species specialisation (d'), network specialisation (H2'), interaction diversity and robustness of plant-frugivore networks (n = 9) in relation to fruit abundance, fruiting plant species richness, canopy cover (%) and season (2009/10 and 2010/11). A subset of the complete data set was used for these analyses. For each species where more than 6 h of observation in each year were available, we equally sampled a 6 h-subset of the dataset from the three observation sessions (see main text for details). Effects on all dependent variables were analysed using linear mixed effect models, with year nested within plot. To investigate effects on species specialization, trophic level was nested within year and plot. Given are t and P values. Note: all significant or marginally significant P values are highlighted in bold. (DOCX) [file pone.0054956.s002.docx]

**Table S1.**  **Effects of habitat characteristics and season (year 2009/10, year 2010/11) on the structure of plant-frugivore networks.**

|  | species specialization (*d'*) | | network specialization (*H_2_'*) | | interaction diversity | | network robustness | |
| --- | --- | --- | --- | --- | --- | --- | --- | --- |
|  | t | P | t | P | t | P | t | P |
| fruit abundance | 0.99 | 0.367 | 0.99 | 0.367 | 0.51 | 0.633 | 0.42 | 0.694 |
| fruiting plant species richness | -1.01 | 0.359 | -1.11 | 0.318 | 0.52 | 0.628 | 0.87 | 0.426 |
| canopy cover | -0.12 | 0.913 | -0.52 | 0.628 | 1.14 | 0.307 | 0.18 | 0.863 |
| Year | -0.23 | 0.825 | 0.11 | 0.914 | 1.54 | 0.162 | 0.97 | 0.362 |
| Trophic level | 5.82 | **<0.001** |  |  |  |  |  |  |

Species specialisation (*d'*), network specialisation (*H_2_'*), interaction diversity and robustness of plant-frugivore networks (n = 9) in relation to fruit abundance, fruiting plant species richness, canopy cover (%) and season (2009/10 and 2010/11). A subset of the complete data set was used for these analyses. For each species where more than 6 h of observation in each year were available, we equally sampled a 6 h-subset of the dataset from the three observation sessions (see main text for details). Effects on all dependent variables were analysed using linear mixed effect models, with year nested within plot. To investigate effects on species specialization, trophic level was nested within year and plot. Given are *t* and *P* values. Note: all significant or marginally significant *P* values are highlighted in bold.
