# Supplementary material for: Habitat Characteristics of Forest Fragments Determine Specialisation of Plant-Frugivore Networks in a Mosaic Forest Landscape
Source: PLoS One. 2013 Jan 24;8(1):e54956. doi: 10.1371/journal.pone.0054956 (PMC3554686; doi:10.1371/journal.pone.0054956)
Supplement: Table S2 — Common and scientific names of fleshy-fruiting plants in nine plant-frugivore networks. (DOCX) [file pone.0054956.s003.docx]

**Table S2:** **Common and scientific names of fleshy-fruiting plants in nine plant-frugivore networks.**

| **Common name** | **Scientific name** |
| --- | --- |
| African False-currant | *Allophylus africanus* |
| Tassel-berry | *Antidesma venosum* |
| Mitzeeri Sweetberry | *Bridelia micrantha* |
| White-stinkwood | *Celtis africana* |
| Tinderwood | *Clerodendrum glabrum* |
| Forest Croton | *Croton sylvaticus* |
| Forest Corkwood | *Commiphora woodii* |
| Acorn Jackal-berry | *Diospyros natalensis* |
| Common Wild Fig | *Ficus burkei* |
| Red-leaf Fig | *Ficus ingens* |
| Brown-cluster Fig | *Ficus sur* |
| Wild-plum | *Harpephyllum caffrum* |
| False-assegai | *Measa lanceolata* |
| Black Bird-berry | *Psychotria capensis* |
| Red-beech | *Protorhus longifolia* |
| Wild Date Plum | *Phoenix reclinata* |
| Cheesewood | *Pittosporum viridiflorum* |
| Quinine-tree | *Rauvolfia caffra* |
| Cape-beech | *Rapanea melanophloeos* |
| Waterberry | *Syzygium cordatum* |
| Jumping-seed Tree | *Shirakiopsis elliptica* |
| Common Wild Currant | *Searsia pyroides* |
| Blunt-leaf Crow-berry | *Searsia rehmanniana* |
| Red Currant | *Searsia chirindensis* |
| Forest Natal Mahogany | *Trichilia dregeana* |
| Wild-mulberry | *Trimeria grandifolia* |
| Pigeonwood | *Trema orientalis* |
| Brides-bush | *Tarrenna pavettoides* |
| White-ironwood | *Vepris lanceolata* |
| Small Knobwood | *Zanthoxylum capense* |
| Forest Knobwood | *Zanthoxylum davyi* |
