# Supplementary material for: Habitat Characteristics of Forest Fragments Determine Specialisation of Plant-Frugivore Networks in a Mosaic Forest Landscape
Source: PLoS One. 2013 Jan 24;8(1):e54956. doi: 10.1371/journal.pone.0054956 (PMC3554686; doi:10.1371/journal.pone.0054956)
Supplement: Table S3 — Species codes, common and scientific names of frugivores (birds) in nine plant-frugivore networks. (DOCX) [file pone.0054956.s004.docx]

**Table S3:** **Species codes, common and scientific names of frugivores (birds) in nine plant-frugivore networks.**

| **Common name** | **Scientific name** |
| --- | --- |
| African Green-pigeon | *Treron calvus* |
| African Olive-pigeon | *Columba arquatrix* |
| Barratt's Warbler | *Bradypterus barratti* |
| Black-bellied Starling | *Lamprotornis corruscus* |
| Black-collard Barbet | *Lybius torquatus* |
| Black-headed Oriole | *Oriolus larvatus* |
| Brimstone Canary | *Crithagra sulphuratus* |
| Bush Blackcap | *Lioptilus nigricapillus* |
| Cape Glossy Starling | *Lamprotornis nitens* |
| Cape Turtle-dove | *Streptopelia capicola* |
| Cape Weaver | *Ploceus capensis* |
| Cape White-eye | *Zosterops virens* |
| Cape Robin-chat | *Cossypha caffra* |
| Chorister Robin-chat | *Cossypha dichroa* |
| Collared Sunbird | *Hedydipna collaris* |
| Crowned Hornbill | *Tockus alboterminatus* |
| Dark-backed Weaver | *Ploceus bicolor* |
| Dark-capped Yellow Warbler | *Chloropeta natalensis* |
| Dark-capped Bulbul | *Pycnonotus tricolor* |
| Olive Sunbird | *Cyanomitra olivacea* |
| Forest Canary | *Crithagra scotops* |
| Garden Warbler | *Sylvia borin* |
| Green Wood-hoopoe | *Phoeniculus purpureus* |
| Grey Sunbird | *Cyanomitra veroxii* |
| Knysna Turaco | *Tauraco corythaix* |
| Lemon Dove | *Aplopelia larvata* |
| Southern Double-collared Sunbird | *Cinnyris chalybeus* |
| Malachite Sunbird | *Nectarinia famosa* |
| Olive Thrush | *Turdus olivaceus* |
| Orange Ground-thrush | *Zoothera gurneyi* |
| Red-eyed Dove | *Streptopelia semitorquata* |
| Red-fronted Tinkerbird | *Pogoniulus pusillus* |
| Red-capped Robin-chat | *Cosspypha natalensis* |
| Red-winged Starling | *Onychognathus morio* |
| Scarlet-chested Sunbird | *Chalcomitra senegalensis* |
| Sombre Greenbul | *Andropadus importunus* |
| Southern Black Tit | *Parus niger* |
| Speckled Mousebird | *Colius striatus* |
| Spectacled Weaver | *Ploceus ocularis* |
| Streaky-headed Seedeater | *Crithagra gularis* |
| Tambourine Dove | *Turtur tympanistria* |
| Terrestrial Brownbul | *Phyllastrephus terrestris* |
| Thick-billed Weaver | *Amblyospiza albifrons* |
| Trumpeter Hornbill | *Bycanistes bucinator* |
| Village Weaver | *Ploceus cucullatus* |
| Violet-backed Starling | *Cinnyricinclus leucogaster* |
| White-starred Robin | *Pogonocichla stellata* |
| Willow Warbler | *Phylloscopus trochilus* |
| Yellow Canary | *Crithagra flaviventris* |
| Yellow-rumped Tinkerbird | *Pogoniulus bilineatus* |
| Yellow Weaver | *Ploceus subaureus* |
| Yellow-fronted Canary | *Crithagra mozambica* |
| Yellow-streaked Greenbul | *Phyllastrephus flavostriatus* |
