# Supplementary material for: Habitat Characteristics of Forest Fragments Determine Specialisation of Plant-Frugivore Networks in a Mosaic Forest Landscape
Source: PLoS One. 2013 Jan 24;8(1):e54956. doi: 10.1371/journal.pone.0054956 (PMC3554686; doi:10.1371/journal.pone.0054956)
Supplement: Table S4 — Measures of plant-frugivore networks and habitat characteristics of nine scarp forest fragments in South Africa. (DOCX) [file pone.0054956.s005.docx]

**Table S4: Measures of plant-frugivore networks and habitat characteristics of nine scarp forest fragments in South Africa**.

| Plot ID | Network size  (# frugivore species, # plant species) | Network measures | | | | | Habitat characteristics | | |
| --- | --- | --- | --- | --- | --- | --- | --- | --- | --- |
|  |  | Frugivore specialization (*d’*) | Plant specialization  (*d’*) | Network specialization (*H_2_’*) | Interaction diversity | Network robustness | Fruit abundance | Fruiting plant species richness | Canopy cover |
| 1 | 19,9 | 0.36 | 0.40 | 0.52 | 3.33 | 0.47 | 48199.17 | 9 | 86.75 |
| 2 | 27,11 | 0.31 | 0.41 | 0.42 | 3.67 | 0.48 | 39930.71 | 12 | 91.25 |
| 3 | 25,14 | 0.32 | 0.33 | 0.36 | 3.80 | 0.48 | 63253.41 | 15 | 90.00 |
| 4 | 30,12 | 0.35 | 0.40 | 0.42 | 3.69 | 0.48 | 54085.83 | 12 | 86.25 |
| 5 | 31,13 | 0.34 | 0.39 | 0.42 | 3.84 | 0.48 | 49598.33 | 13 | 76.01 |
| 6 | 23,11 | 0.42 | 0.53 | 0.53 | 3.29 | 0.48 | 66197.57 | 11 | 63.75 |
| 7 | 25,11 | 0.32 | 0.31 | 0.39 | 3.27 | 0.48 | 10759.00 | 12 | 80.50 |
| 8 | 24,11 | 0.34 | 0.38 | 0.44 | 3.60 | 0.48 | 30883.92 | 11 | 92.00 |
| 9 | 18,11 | 0.33 | 0.37 | 0.43 | 3.38 | 0.47 | 40180.80 | 11 | 90.50 |
